# Navigating transitions into, through, and beyond peer worker roles: insider insights from the Supporting Harm Reduction through Peer Support (SHARPS) study

**DOI:** 10.1186/s12954-024-01109-4

**Published:** 2024-10-28

**Authors:** Josh Dumbrell, Hannah Carver, Rebecca Foster, Bernie Pauly, Wez Steele, Michael Roy, Tessa Parkes

**Affiliations:** 1 Salvation Army Centre for Addiction Services and Research, Faculty of Social Sciences, University of Stirling, Stirling, UK; 2 School of Applied Sciences, Edinburgh Napier University, 9 Sighthill Court, Edinburgh, UK; 3 Canadian Institute for Substance Use Research, University of Victoria, Victoria, Canada; 4 Scottish Drugs Forum, 91 Mitchell Street, Glasgow, UK; 5 New Vision Bradford, Humankind, Pelican House, 10 Currer Street, Bradford, UK

**Keywords:** Substance use, Homelessness, Complex needs, Peer workers, Experiential workers, Workers with lived and living experience, Harm reduction, Psychologically informed environments, Qualitative

## Abstract

**Background:**

Peer workers are individuals who draw on their personal experiences in a professional capacity to support clients. Existing research on the role of peer workers in mental health, homelessness, and substance use services has primarily focused on their impact on client outcomes. This paper describes the development of peer workers as they transition into, through, and beyond this role. Utilising data from the Supporting Harm Reduction through Peer Support (SHARPS) study, where Peer Navigators supported people experiencing homelessness and substance use challenges, this paper explores the sense-making involved in an intensive peer support worker role, adaptation to organisational culture, and engagement with opportunities for professional advancement.

**Methods:**

Semi-structured interviews with three Peer Navigators were conducted by two SHARPS study researchers at four time points in 2018 and 2019 corresponding with the beginning, middle, and end of the intervention. These data were analysed along with entries from the three Peer Navigators’ reflective diaries. Analysis followed a multi-stage approach to thematic analysis utilising both inductive and deductive processes. The Peer Navigators’ personal reflections have also been incorporated into the recommendations.

**Results:**

The foundational training provided to the Peer Navigators before taking up their role helped to ensure readiness and build confidence. This training illuminated the dynamics of supporting individuals with complex health and social challenges. Integrating into diverse organisational environments, the Peer Navigators adapted to new professional expectations and consistently advocated for harm reduction and psychologically informed approaches, sometimes encountering resistance from other professionals. Establishing effective relationships with participants and professionals was essential and involved dealing with challenges such as overcoming personal biases and navigating systemic obstacles. the Peer Navigators benefitted from the specially designed training to support career progress with personal and professional development opportunities which enabled successful transitions beyond the SHARPS study.

**Conclusions:**

Pre-work training, coupled with support and adherence to key principles, enabled the Peer Navigators to integrate effectively into diverse organisations. Quality relationships were vital in achieving client outcomes and supporting the professional growth of the Peer Navigators. These findings are important for services employing peer workers and underscore the importance of a commitment to training and continuing professional development.

**Supplementary Information:**

The online version contains supplementary material available at 10.1186/s12954-024-01109-4.

## Background

Peer workers are individuals who leverage their personal experiences of specific issues or conditions to foster the establishment of trusted relationships to support others (clients) facing similar challenges [1]. Previous research has demonstrated the effectiveness of these roles, describing a variety of positive client outcomes, including enhanced engagement, increased confidence and self-efficacy, and a sense of empowerment and hope [2–4]. Indeed, the client outcomes of peer worker-led interventions are a key focus within the literature on mental health [1, 3], homelessness [5], and substance use [6]. Most specifically, research at the intersection of homelessness and substance use has explored the experiences of peer workers themselves, particularly the challenges they face [7]. However, insights into the development of peer workers as they transition into, through, and also beyond their roles are limited. Some studies have explored elements of the peer worker experience that broadly reflect personal and professional transition milestones. Such transitory markers include orientation and training [8–10], adapting to organisational cultures [9], managing challenges [11], and engaging with organisation-led support, self-care practices, and opportunities for personal and professional development [8]. Understanding peer worker transitions is crucial for tailoring effective support systems, enhancing service delivery, fostering professional growth, and developing and valuing the lived experience workforce within organisations. Recognising the transitions that individuals go through also promotes inclusivity, bolsters knowledge, and facilitates a more comprehensive and adaptable approach to employing experiential workers.

Exploration of peer worker training shows that, while most undergo some specialised training, including professional boundaries and appropriate disclosure [5, 7, 8], there exists significant variation in duration, intensity, and content [8, 10, 12, 13]. For example, a recent systematic review of peer work in drug use service settings reported a variety of training, with an overall focus on task-specific knowledge, skills unique to peer services like communication and leveraging lived experiences effectively, and strategies for navigating professional environments including collaboration with clinicians [13]. Indeed, the variable representation of peer worker training described in the peer-reviewed literature arguably overlooks the extensive work documented in the grey literature which can provide richer insights into the area of development and support for peer workers [14]. Of note, some writers have expressed concern that the ‘upskilling’ of the lived experience workforce could inadvertently transfer responsibility from professionally trained practitioners to underqualified peer workers, thereby risking the integrity and effectiveness of peer worker roles [15]. Regardless, the literature on peer worker training, especially within homelessness and substance use settings, could benefit from a broader exploration of the training experiences of peer workers, and its impact on practice.

Organisational culture, values, and practices significantly affect the integration and support of peer workers, with their professional development hinging on adapting to these environments and navigating policies, communication with colleagues, and alignment with organisational goals [16, 17]. The experiential knowledge of peer workers enables them to engage with clients, establish trust, promote inclusivity, and facilitate positive change [16, 17]. Yet peer workers face integration challenges, supervision largely by professionals without lived experience, and role constraints due to the differing value placed on professional versus experiential knowledges [15, 18–21]. Despite the growing recognition of the value of lived experience, concerns persist that organisations prioritise formal education over experiential knowledge [11], and even that professionalisation, per se, may detract from the essence and effectiveness of peer work [8, 15]. Exploring peer workers’ experiences across various settings can thus highlight how organisational cultures and readiness levels impact peer work, offering crucial insights for improving integration, support, and effectiveness, and benefitting both academic research and practice in the field.

While there exists discussion of the positive effects of engaging in peer work such as educational readiness [22], enhanced self-esteem, health management [18], and reduced behavioural risks related to drug use [19], explorations of the nature and extent of professional development opportunities available to peer workers remain limited. While Mamdani et al. [20], for example, acknowledge the diverse training needs of peer workers, encompassing technical (helping people access income assistance), people skills (navigating unpleasant interactions with service providers), and self-care skills, their discussion of training needs did not extend to exploring progression beyond the role. More relevant to the focus of this current paper, Olding et al. [16] identified limited prospects for the advancement of peers by noting the presence of professional barriers. Contrastingly, though Annand and colleagues’ [8] peer homelessness health advocates were employed as volunteers, the authors described a clear pathway to paid employment within the organisation. Of note, these common transitions were facilitated by support from a community of staff members, volunteers, and specialised personnel who offered assistance in various areas such as benefits, wellbeing, and job-seeking which ensured that peers had the necessary support. Due to the paucity of comprehensive investigation into peer worker progression as they transition into and through their roles, this paper aims to bridge this gap by presenting original research data that examines the experiences and trajectories of peer workers, including their pursuit of professional and career development.

The Supporting Harm Reduction through Peer Support (SHARPS) study, conducted between May 2018 to May 2020 in England and Scotland (UK), tested the feasibility of a co-produced peer-led, relational, harm reduction intervention for individuals experiencing homelessness and substance use problems. Four part-time (30 h per week) Peer Navigators provided practical and emotional support to a case load of up to 15 participants, for between 2 and 12 months. They were employed on 18-month contracts by The Salvation Army, at a specialist support worker rate, a rate of pay which reflected their intensive training and role expectations. The time-limited nature of the roles was due to the research grant funding which was for two years. One Peer Navigator left the study early and their data were not included in this paper. Detail regarding the study design and intervention can be found in related publications [4, 21]. This paper draws on data specifically collected from three of the Peer Navigators, who also share authorship of this paper, to understand their experiences over the course of the intervention. All three Peer Navigators provided approval for their data to be used for this paper, with additional ethics approval granted for this purpose.

## Methods

### Aim and design

The aim of this paper is to explore the key experiences and challenges faced by the SHARPS Peer Navigators during their transition into, through, and beyond their roles as peer workers. The time-limited nature of these roles made it ethically important to explore and support transitions into and out of role. The paper examines how the three Peer Navigators navigated and adapted to the demands and expectations of their roles and identifies effective strategies and support systems for their career progression. The paper utilises data from semi-structured interviews with the three Peer Navigators, and insights from 15 of their reflective diaries (kept throughout their 18 month positions) to address research questions 1 and 2 below. Research question 3 relates specifically to outcomes post-study and is addressed through author reflections which supported recommendation development. Because these reflections were not part of the original SHARPS study, they have been articulated as recommendations.

1. What were the key experiences and challenges that the SHARPS Peer Navigators encountered during their transition into role, and during their peer worker roles?
2. How did the Peer Navigators navigate and adapt to the demands and expectations of their roles as they progressed through different stages of their journey?
3. What strategies, support systems, and relationships were effective in facilitating successful transitions and career progression for the Peer Navigators as they moved beyond the SHARPS role?

### Participants and data collection

Semi-structured interviews were conducted with the Peer Navigators by two academic researchers from the SHARPS study team (HC, RF). These were undertaken at four time points (June/July 2018, April 2019, June 2019, and November 2019) in order to capture changes in perception of their roles across the early and midway periods, and then at the end of the intervention. The aim of these interviews was to understand Peer Navigator experiences and views of the intervention, as well as changes to their practice and knowledge. The interviews were conducted by phone or in person. Interview schedules (Supplementary File 1) included questions about experiences of the intervention, perceptions of the role, past experiences of working in services, differences between the SHARPS intervention and current practice, experiences of training, challenges experienced, and changes to practice while in role. The Peer Navigators provided written informed consent to participate in the study and verbal consent was checked prior to each interview. Reflective diaries were kept for them to share their feelings/reflections while they were in the role.

### Data analysis

Interviews and reflective diaries were transcribed and analysed by JD, using Braun and Clarke’s [23] approach to thematic analysis and incorporated both inductive and deductive techniques. Initial transcript readings generated preliminary notes, before undertaking line-by-line coding using NVivo 12 (QSR International, 1999). Codes were organised into potential themes by JD, reviewed for consistency, and then verified by HC. Deductive analysis [24] refined the themes (JD), aligning them with the first two research questions and existing literature. In the final stage, theme-exemplifying extracts were selected (JD), presented with quotations, and are reported below.

### Findings

A total of four themes and eight sub-themes are presented below.

**Table 1:** Themes, sub-themes, and descriptions

Themes	Sub-themes	Description
Making sense of the role	Flexibility and boundaries Harm reduction ethos	Explores Peer Navigators’ transitions into a complex, multifaceted role, and their embracing of harm reduction.
Building capacity and adapting to organisational culture	Training – for role effectiveness and the future Settings, intervention buy-in, and support	Details Peer Navigator experiences of the foundational training and delivering the intervention across different settings.
Managing relationships and building professional networks	Peer Navigator-client relationships Developing professional networks for effective care	Focuses on establishing and maintaining relationships with clients and colleagues.
Professional development opportunities	‘Soft’ skill development Practical skill acquisition and application	Highlights skill acquisition and development supporting role transitions.

The theme ’Making sense of the role’ offers a nuanced exploration of Peer Navigators’ evolving understandings of their multi-faceted roles and underpinning ethos. ‘Building capacity and adapting to organisational culture’ represents two other key transitions, as Peer Navigators detail their experience of intensive training and delivering the intervention across distinct settings, with variable degrees of buy-in and experiences of support. In many ways central to the role, ‘managing relationships and building professional networks’ outlines the invaluable process of establishing and maintaining relationships with clients and colleagues. Lastly, ‘professional development opportunities’ highlights skill acquisition and personal and professional development which were acknowledged by the Peer Navigators as preparing and supporting their shifts through and beyond peer worker roles. The selection of themes reflects those areas of discussion which concern the dynamics of transitioning into and through peer worker roles, with a key focus on role development, support, and onward advancement (Table 1).

### Making sense of the role

From initial diverse expectations to becoming advocates within complex systems, Peer Navigator journeys were marked by necessary adaptation and continuous growth. Broadly, the sense-making process converged around two central sub-themes, flexibility and boundaries, and an emergent harm reduction ethos which came to underpin understandings and execution of their role.

### Flexibility and boundaries

From the outset, Peer Navigators faced an array of expectations. They assumed multifaceted roles – supporting clients as mentors, confidants, and advisors, while advocating for those struggling within inflexible and punitive service structures. The diversity of responsibilities necessitated significant flexibility as they were often required to adjust their approaches depending on the client and their immediate needs. The Peer Navigators reflected on the diverse ways they could support individuals, emphasising how this flexibility helped them to bridge gaps between services.

*I think almost like a mentor*,*in a kind of sense*,*or*,*it’s going to be a lot of things… buddy*,*confidant*,*it’s going to be*,*point of information*,*it’s going to be advice*,*it’s going to be guidance*,*it’s going to be sounding board*,*it’s going to be making someone a cup of tea who is freezing cold and piss wet through*,*it’s going to be all of the above really.* (Interview 3)

A recurring theme was a struggle to define the contours or boundaries of their roles. In addition to adapting to the intervention’s operational demands, the Peer Navigators grappled with an internal conflict, feeling like imposters in their own domain:

*I’ve found it a little bit challenging in the earlier stages defining my own role and defining what my role is and what it isn’t… almost feeling like an imposter*,* or feeling like I shouldn’t say this*,* or I shouldn’t do that.* (Interview 11)

Despite these insecurities, there was an acknowledgment that the inherent flexibility of their role was advantageous because it could allow unconventional approaches at times and be completely tailored to individuals, many of whom did not fit neatly into office hours, though important work still had to done.

*It’s having someone that can work*,* work personally with a person like that*,* be flexible enough to be able to work*,* yeah work to their schedule to a degree* (*…*) *about knowing when to*,* where the boundaries must lie and if we are going to achieve anything there has to be some sort of rules.* (Interview 2)

Similarly, the scope of the role was at times difficult to define, though all Peer Navigators reflexively managed boundaries while noting that this was often a collaborative effort with clients:

*Things were going alright and then s/he called me at five o’clock this morning as well*,* 5.20*,* just literally at the time that my alarm goes off and sent about ten texts*,* so I think maybe s/he is on one*,* we need to have a talk about boundaries.* (Interview 8)

While this adaptability and flexibility was a key strength, it also posed challenges. The breadth of their responsibilities often left the Peer Navigators grappling with blurred boundaries, particularly when they felt compelled to compensate for the failings of other services. For example, at times they took on tasks beyond their formal remit, feeling pressure to advocate for clients when services were perceived as slow or unresponsive. This led to a realisation among the Peer Navigators that, while they needed to be flexible to address immediate client needs, they also had to set limits to avoid taking on too much and preventing other agencies from fulfilling their roles:

*It felt*,* and still feels like*,* the other agencies involved are not really doing anything they are meant to be doing… so your point here is to chase them*,* as much as it is to do the bit you can do. Don’t take on doing everything.* (Interview 9)

Among these considerations was Peer Navigator closeness to client experience and appropriate disclosure, which demanded dynamic boundary management, in the context of a peer to peer relationship, to ensure vital harm reduction messages were received:

*I am bigging up on the hand washing and just highlighting that there is a lot of infections and that they are quite serious and I can disclose*,* well I suppose fortunate is not the right word but I’ve got something I can say look I didn’t do some of that stuff and this is what happened to me*,* but I do it in a way not to say oh you will end up like me because they won’t it could be totally different*,* but to just highlight and say for the sake of taking two minutes to wash your hands you could potentially not end up in hospital.* (Interview 5)

Actively managing the balance of flexibility and boundaries was a major theme in Peer Navigator reflections. They spoke about the importance of being flexible to align with the unpredictable schedules and needs of their clients but also acknowledged the need for boundaries if meaningful progress was to be made. This careful negotiation of when to step in, when to step back, and when to disclose, became a key part of their role as they adapted to the operational demands of the intervention. Over time, they became more confident in managing this balance, acknowledging that while flexibility was essential it had to be grounded in clear boundaries to maintain the integrity of their work.

### Harm reduction ethos

The Peer Navigators reflected on the evolution of their roles and perspectives. One consistent transformation across the three Peer Navigators concerned the integration and acceptance of a harm reduction-focused approach, despite initial perceptions of a “*very sharp divide between recovery and harm reduction*” (Interview 12). Characterising this re-evaluation, one Peer Navigator defined his approach as a nuanced blend of harm reduction and other soft skills for supporting positive outcomes among those on his caseload:

*When I first started*,* I thought it was all about getting people to stop or reduce using* [drugs/alcohol] *which is some of the focus*,* some of the time*,* as appropriate after all the soft skills stuff has taken place and a lot of the time it’s not. And it’s just managing that and being alright with that.* (Interview 11)

Two of the three Peer Navigators initially described uncertainty and perceived tension between harm reduction and the more personally and professionally familiar abstinence-focused approach. However, given the admission that “*probably 99% of what my role will be harm reduction* (Interview 2), they acknowledged the need to adapt to embedding harm reduction practices and principles into the delivery of their roles. One Peer Navigator noted their prior experience in an abstinence-based residential treatment setting and anticipated the challenges of transitioning to a harm reduction approach:

*So*,* the only job I’ve done in health and social care settings is very in line with my* [personal] *recovery model. Does that make sense? This is going to be very different… yeah it’s going to be very different doing harm reduction isn’t it?* (Interview 3)

Another reflected on the historical conflict between recovery services and harm reduction services, highlighting the necessity to accept harm reduction fully:

*I was expecting a bit of conflict as a person in recovery*,* and throughout my recovery there has been this kind of conflict between these services*,* like harm reduction services and abstinence… I am certainly being brought around to the idea because*,* in the simplest terms*,* everyone’s journey*,* or most people’s journeys begin with harm reduction*,* and so yeah*,* it’s just something that I am going to have to*,* I am going to have to accept.* (Interview 2)

These initial interviews captured the Peer Navigators’ developing appreciation of, and adaptation to, a harm reduction ethos. Over time, the Peer Navigators’ understanding of harm reduction evolved from viewing it as a set of specific practices to embracing it as a more comprehensive philosophy. This shift occurred as they recognised the relational nature of harm reduction and its broader applications, which became more intuitive in their practice. For example, trust, and building rapport through non-judgment were identified as key components in addressing harmful behaviours. The importance of appropriate communication was also stressed, acknowledging the relevance of relationships and rapport to harm reduction conversations:

*If you build a relationship or build a rapport with someone you are doing the job. If you are talking about reducing a damaging behaviour you are doing your job.* (Interview 4)

Experience in the role helped Peer Navigators transition from a basic understanding of harm reduction practices, such as advising clients to smoke rather than inject, to a more comprehensive and nuanced grasp of the philosophy underlying harm reduction:

*Yes*,* tell people to smoke it like but that’s not effective harm reduction in my opinion*,* because they know if they are getting abscesses or if they are doing damage*,* they know that they are causing harm in the behaviour they are doing so it’s always just getting the right little fit or the right little step or opening the conversation in the right way*,* would you be open to trying to – do you know what I mean?* (Interview 4)

Initially, the emphasis was on pragmatic, practice-based interventions, such as providing safer alternatives to injecting. However, over time, Peer Navigators began to appreciate that harm reduction is not just about these surface-level practices but is grounded in a deeper understanding of the broader context – one that includes the history of drug prohibition, unequal power dynamics, stigma, and the importance of fostering trust. The Peer Navigators’ understanding of harm reduction evolved significantly over time. Initially, they viewed it as a set of specific practices, often in tension with abstinence-based approaches. However, through their experiences, they began to recognise harm reduction as a more comprehensive philosophy, grounded in fostering trust, autonomy, and non-judgment. This shift enabled them to move beyond surface-level interventions, like advising safer drug use, to helping address broader issues such as stigma, the criminalisation of substance use, and power dynamics. By the end of the intervention, their deeper understanding of harm reduction had enhanced their ability to build rapport, communicate effectively, and support clients with empathy and respect for their independence.

### Building capacity and adapting to organisational cultures

The effectiveness of Peer Navigators in delivering interventions relied not only on their personal and professional development but also on their ability to adapt to the organisational cultures within which they worked. The focus on building capacity through targeted training and adapting to varying levels of organisational support was crucial for Peer Navigators to effectively fulfil their roles and pursue future career opportunities. While they benefitted from structured training and support, the success of their work depended on their capacity to adjust to service environments, manage resistance, foster collaboration, and negotiate change within often rigid, traditional systems.

### Training – for role effectiveness and the future

Peer Navigators underwent three months of intensive training relevant to the role. Their training included participatory workshops, seminars, and short courses covering harm reduction, the impact of trauma on substance use, professional boundaries, therapeutic relationships, and Psychologically Informed Environments (PIEs) – an approach involving local, reflective initiatives that enhance understanding of and responses to, service clients’ psychological and emotional needs [25], along with study-specific training on recruitment, ethics, eligibility assessment, and informed consent. Training was regarded as central for Peer Navigators, serving as the bridge between effective current practice and future career progression. Reflecting on their experiences, the Peer Navigators acknowledged the foundational role of training in honing their skills for client engagement and personal growth.

The Peer Navigators reflected fondly on the induction and early training period, which was regarded by one as well-structured, while the range of courses were seen as vital, both for supporting relational work with clients, and for aiding in the delivery of effective research for the SHARPS study.

The Peer Navigators reflected upon attending specific workshops on subjects like complex trauma and gender-based violence, which, while challenging, were considered essential for enhancing understanding and empathy:

*I actually enjoyed*,* in a weird way*,* the complex trauma training which was focused around gender-based violence that we went to in Glasgow. I found it a very difficult subject matter to sit through and listen to*,* but very beneficial and informative for some of the people that I’ve been working with.* (Interview 11)

The Peer Navigators indicated that some training courses were immediately applicable in practice, others addressed gaps in knowledge while ongoing reflective practice at times shifted the Peer Navigators’ approach to their work:

*So*,* the motivational interviewing was just like*,* it made me realise that they had to sort of reach their goals and targets*,* and I mean*,* not me trying to take everything and achieve it all do you know what I mean*,* do everything for them.* (Interview 6)

Amidst the transitory nature of their roles, the Peer Navigators were prompted to seek training opportunities to prepare for future employment and ease the inherent anxieties of contract work. They viewed training as a vital component of their professional development, positioning them strongly for future endeavours. Whether it was understanding aspects of the health, social care, and welfare systems or gaining knowledge in other domains, all Peer Navigators showed a keen interest in harnessing training opportunities, valuing each learning experience as indispensable for their professional journeys:

*It’s just professional development*,* I suppose a lot of the training we are going to be getting is going to be really good anyway*,* and that’s just stuff that occurs to me with a view to what happens when the study is finished to kind of put myself in a stronger position*,* I suppose*,* professionally going forward.* (Interview 3)

Striking a balance between personal- and client-oriented development was also emphasised, reflecting awareness of the importance of growth within and beyond the project. The accessibility of external training was particularly valued as it offered flexibility and a chance to further the Peer Navigators’ knowledge and skills:

*I’ve loved the fact that we are able to and supported to find and attend external training if we want… you want to take the opportunities from a professional development and a personal development point of view but what’s maybe best for the client base is striking that balance and sometimes saying ‘thank you for the offer but I would be better served doing this*,* this and this’.* (Interview 11)

The Peer Navigators recognised the dual significance of training: it not only improved their immediate work with clients but also equipped them for future professional challenges. Emphasising both personal and client-oriented development, they viewed continuous learning as a strategic step toward professional resilience and advancement.

### Settings, intervention buy-in, and support

Navigating the nuances of different statutory and third-sector organisational cultures presented a multifaceted challenge for Peer Navigators, who encountered a spectrum of responses to their roles, from broad acceptance to scepticism regarding the presumed ‘added value’ of peer-led support work. The adaptation to varying degrees of intervention buy-in and the vital quest for support across varying health and social care landscapes marked a significant aspect of each of their journeys.

Despite some supportive environments (i.e., within host institutions, and alongside the SHARPS research team), Peer Navigators faced significant challenges, including resistance and cynicism in various settings, due to role overlap and inconsistent adoption of harm reduction principles. These issues highlighted the difficulties of integrating new interventions into established healthcare systems.

The Peer Navigators expressed feeling “*well looked after*” (interview 2) throughout their roles, including benefitting from practical and emotional support from a range of people. They also noted being able to speak openly and honestly in their host service:

*Yes*,* I feel really well supported in my main host service… I feel like I can be very open and honest*,* and I can be wrong*,* but I can still voice my opinion.* (Interview 4)

Despite the multidisciplinary nature of the role, the Peer Navigators reflected on the comfort they felt with colleagues on the research team and the sense of professional equality:

*I don’t feel so much of a professional difference*,* if that makes sense.* (Interview 3)

Some professionals in other services displayed resistance to the Peer Navigators, however, questioning the support they offered and sometimes stating that it encroached on their responsibilities. This resistance led to feelings of discomfort and feeling a little unwelcome in certain service settings:

*Some professionals in other services have given the impression that they think me fulfilling my role is somehow encroaching on them fulfilling their role*,* or that I shouldn’t be offering certain types of support because they offer it.* (Interview 4)

The Peer Navigators’ overt adherence to a harm reduction ethos and the principles of PIEs at times stood in contrast to the established practices evident in some service contexts, where acceptance of the novel intervention was perceived as less than desirable:

*If you are working in a psychologically-informed environment I think that’s why you need that bit more attentive support if you are working at that model*,* because if we are not*,* if we are not judging people by their behaviour but trying to understand it I think that takes a lot more work and we’d be sat with that client a lot more and understanding them and the backgrounds and everything of why somebody is maybe behaving in that way.* (Interview 6)

The collaboration between the Peer Navigators and other professionals varied significantly. While some external services presented challenges, interactions with certain individuals, like drug liaison nurses, were highly positive and productive, facilitating effective coordination and support for clients:

*I feel it’s been beneficial having the drug liaison nurse as a point of contact within the hospital for times when I’ve needed to get stuff done on people’s behalf… because we’d be lost without her really*,* I would be no one*,* just a guy from the Salvation Army but she’s managed to get things done. And at least it’s reassuring certainly for one guy who’s in a bad way physically and emotionally being in hospital*,* so it’s reassuring to know that we are all able to link up.* (Interview 5)

The Peer Navigators often found themselves at the intersection of support and resistance within the organisational culture of some statutory services. While some environments provided a strong sense of belonging and support, others manifested resistance, highlighting the complexity of integrating progressive harm reduction strategies into traditional settings. The success of such integration relied heavily on the collaborative efforts and open-mindedness of dedicated professionals across the spectrum of care.

### Managing relationships and building professional networks

Managing relationships and building professional networks was a pivotal aspect of the Peer Navigator role. The strength and depth of these relationships were reportedly not only crucial for client engagement and support but also for the creation of a collaborative professional environment that enhances service delivery and intervention outcomes.

### Peer Navigator-client relationships

The Peer Navigators placed significant emphasis on developing meaningful relationships with clients, recognising this as a core element of their intervention. They found that building rapport was often easier and more reciprocal than anticipated, contributing to successful client outcomes.

Despite this being a core element of intervention design, the Peer Navigators each recounted multiple examples of the surprising ease with which they built relationships with clients. One Peer Navigator shared how his asset-based approach encouraged reciprocal banter from clients, in contrast to the often problem-centred interactions clients can face elsewhere:

*I think that you can build a relationship within an hour*,* with somebody*,* quite a positive relationship… I try to just go in and build that relationship around ‘what are your enjoyments in life*,* what are your interests?’ Have a laugh with them*,* have a bit of banter*,* and let the relationship grow from there.* (Interview 6)

The other two Peer Navigators articulated how their approach had enabled a process among some clients of gradually lessening resistance to the idea of working with them:

*Little signs of [a] guard coming down with one or two clients probably in each service.* (Interview 8)

Reflecting upon client feedback describing some relational qualities of the Peer Navigator intervention, the same Peer Navigator highlighted compassion and empathy as distinct characteristics:

*When I speak to them this is their words coming from them*,* that they don’t see other members of staff have that compassion or have that empathy around what they are going through*,* they are judged.* (Interview 6)

Alongside emotional support, the Peer Navigator also explained that the practical assistance provided in the context of Peer Navigator-client relationships had the capacity to invoke feelings of gratitude and respect from clients:

*It’s been great having I guess the genuine relationships with people and feeling that they respect me for the most part. And that they appreciate the work that I’ve done.* (Interview 12)

The capacity of Peer Navigators to foster trust and respect through compassionate and empathetic engagement was instrumental in decreasing client resistance and promoting positive change. The relationship-building aspect of their role was highlighted as equally beneficial for clients and fulfilling for the Peer Navigators themselves.

### Developing professional networks for effective care

The impact of the Peer Navigators extended beyond client interactions to include the strategic development of professional networks and collaborations. These connections were fundamental in providing comprehensive support for clients with complex needs. One Peer Navigator positioned relationships as the central mechanism driving intervention efficacy:

*It is all about the relationship but also it really is all about the relationship*,* if that makes sense*,* the study is about the relationship but also us achieving some stuff and doing some work together.* (Interview 5)

Such professional networks were understood at the outset as being essential for enabling joined-up support for individuals with complex needs. One Peer Navigator noted that attention to developing professional relationships was a key practical aspect of effective support work and thus a vital feature early in the project’s delivery:

[Local practitioner and SHARPS team member] *sent around that email to everyone in the world and then we went on loads of visits.* (Interview 5)

While acknowledging the importance of early network development, local integration, recognition, and acceptance of the Peer Navigator intervention were reportedly a gradual process:

*Yes*,* getting quite steady referrals from them* [drug liaison nurses] *actually and she is very good at following up and keeping me posted.* (Diary 4)

An example of this developing trust among providers can be seen below, where a Peer Navigator notes their role in facilitating policy shifts to the benefit of clients seeking access to drug treatment:

*Allowances have been made recently*,* to get people on*,* like we were able to get people on*,* straight on scripts*,* same day*,* me and [other Peer Navigator] both did it on the same day*,* which was awesome.* (Interview 5)

Likewise acknowledging the significance of intervention design and early communications for developing connections across the respective health and social care landscapes, another Peer Navigator noted the capacity of the SHARPS study team to influence outcomes and effect change:

*I know you guys [study team] obviously have a bit of clout with getting things rushed through that may be needed.* (Interview 2)

As identified above in ‘settings, intervention buy-in, and support’, professional experience was also identified by all Peer Navigators as allowing them to make informed decisions regarding which other professionals were best to approach for support:

*I know who to approach*,* which staff members are going to be the most… there are people I go to more often than other people if I need advice.* (Interview 5)

Lastly, and foreshadowing the following discussion of professional development, one Peer Navigator highlighted that the specific connections made while undertaking his work had helped guide and reinforce his future career aspirations:

*Meeting the people I’ve met*,* and the more that I think about what I might want to do in the future now it does seem a lot of it has been driven by getting this job and people I’ve met.* (Interview 5)

The gradual cultivation of trust within the professional community allowed Peer Navigators to benefit from policy changes and navigate systems more effectively for their clients. These networks not only empowered the Peer Navigators to enact immediate support but also informed and inspired their future career trajectories.

### Professional development opportunities

This theme tracks the progression and career advancement of the Peer Navigators within and beyond the SHARPS study, emphasising their development in areas like confidence, interpersonal capabilities, academic engagement, and practical skills.

### ‘Soft’ skill development

The Peer Navigators’ accounts track their individual trajectories of personal and professional growth. While initial challenges like social anxiety and imposter syndrome were noted, the Peer Navigators increasingly enhanced their ‘soft’ skills (e.g., communication, empathy, teamwork, and problem-solving abilities) through embracing opportunities such as speaking at conferences and facilitating multiagency meetings. Such participation was described as transformative, boosting confidence and assertiveness:

*Coming to the conferences has been a great experience… good for my own personal development of giving me confidence.* (Interview 10)

*For the last six months… I’ve been a lot more proactive and assertive at multiagency meetings.* (Interview 11)

These accounts reflect a positive and meaningful shift from initial hesitancy to more proactive and confident participation in professional settings.

Likewise, and linked to the theme of ‘managing relationships and building professional networks’ above, the Peer Navigators described the development of interpersonal skills, honed through interactions with clients and colleagues alike. They reflected upon the value of employing active listening, with one practicing the skill of ‘holding space’ within emotionally charged support contexts:

*Just sitting with someone who is obviously suffering quite a bit and just going ‘do you know what I will sit with you while you feel like shit’.* (Interview 4)

Again, practicing understanding, empathy and non-judgement were recognised as the essential conditions for effective relational work.

The Peer Navigators’ journeys illustrate a transformation from tentative beginnings to becoming proactive, confident professionals, highlighting the profound impact of ‘soft’ skill development on personal empowerment and the capacity to inspire change.

### Practical skill acquisition and application

The SHARPS study provided a fertile ground for the Peer Navigators to acquire practical skills that not only enhanced their current roles but also laid a foundation for future academic and professional endeavours.

One Peer Navigator undertook a Master’s module in homelessness and inclusion health during the course of his employment/the study, finding it to align well with his training for the role, while another expressed interest in furthering health and social care training. These examples highlight the value placed by the SHARPS team in facilitating these pursuits for the Peer Navigators’ progression:

*Every lecture was really good*,* very completely consistent with the training that we’ve had here.* (Interview 5)

The Peer Navigators described acquiring and applying various skills critical to their roles which were often also acknowledged to be highly transferable to future pursuits. Key areas included time management, managing a caseload, harm reduction expertise, and budget management. For instance, one Peer Navigator spoke of juggling familial responsibilities with training courses, indicating the importance of time management. Importantly, participation in the SHARPS study was recognised as transformational, both helping to guide local shifts in practice and ensuring the Peer Navigators received recognition from other agencies and the sector more broadly:

*It’s been very cool to be involved in something that is cutting edge and that will be talked about and that will have an impact.* (Interview 12)

*Someone just invited me to go and speak to him about*,* they are trying to adopt a new way of working*,* yeah*,* and it’s just sometimes it blows me away a bit*,* it’s like ‘are you sure you’ve got the right person?’.* (Interview 9)

The practical skills gained through the SHARPS study, from time management to sector-specific expertise, were acknowledged as invaluable. This experience did not just equip the Peer Navigators with immediate tools for their work but also positioned them for recognition and opportunities in the broader professional sphere, signalling the study’s role as a catalyst for enduring professional growth.

## Discussion

This paper illuminates the dynamic and evolving nature of the roles of the SHARPS study Peer Navigators and unveils the multiple layers of their professional development as they transition into, through, and beyond the role. Initially entering their roles with a wide range of expectations, the Peer Navigators confronted both external challenges, such as scepticism from other professionals, and internal struggles like adapting to flexibility and defining the boundaries of their responsibilities. Over time, these initial challenges gave way to a clearer focus on harm reduction approaches, which was accompanied by increased self-confidence and a shedding of early self-imposed pressures. Training was a pivotal element in this journey, serving not merely as an educational tool but also as a mechanism for personal and professional transformation. As the Peer Navigators progressed through their roles, they shifted from grappling with the complexities of their responsibilities to leveraging their training to address these challenges more effectively. Their interactions with other professionals and services were equally complex, ranging from resistance and scepticism to support and collaboration. Despite challenges gaining full acceptance within existing health and social care systems, the Peer Navigators used these experiences to refine their approaches and strategies, leading to improved client outcomes and professional relationships. Central to their role was the ability to build and maintain strong relationships, both with clients and within a broader professional network. These relationships were not just instrumental in achieving client-focused outcomes but also pivotal in the Peer Navigators’ own professional development, opening doors for future career opportunities. Additionally, the Peer Navigators honed practical skills, such as time management and caseload handling, skills they identified as transferable to future roles. This practical skill acquisition occurred alongside ‘soft’ skills like empathy and active listening, further enriching their professional toolkit, and positioned them well for sustainable progression beyond the project’s duration.

This paper aligns with and diverges from existing literature in critical ways, offering fresh perspectives on the multi-faceted roles of peer workers working at the intersection of substance use and homelessness. For instance, findings on boundaries from this study align with existing literature, which highlights the ongoing negotiation of personal and professional roles among peer workers. Like the Peer Navigators in this study, Tookey et al. [26] found that peer workers had to establish clear boundaries, often requiring personal changes, such as moving to new communities. Similarly, the challenges of selective self-disclosure and balancing rapport with clients were noted both in this study and by MacLellan et al. [27], demonstrating that effective boundary management is key to maintaining professionalism while fostering trust. Additionally, Chapman et al. [28] highlighted the unique struggles of peers in recovery, which this study echoed, showing that Peer Navigators needed to carefully balance empathy and personal recovery with their roles as support workers. This paper adds a deeper understanding of how dynamic boundary management evolves throughout the peer worker role, particularly for those navigating personal recovery alongside professional responsibilities. The harm reduction ethos subtheme is also consistent with findings from Tookey et al. [26], where peer workers faced tension between harm reduction principles and their own abstinence-based recovery. In this study, Peer Navigators struggled with the transition to harm reduction but ultimately adapted, acknowledging its necessity. The emotional strain described by Peer Navigators as they witnessed clients continue to use substances aligns with Tookey et al.’s [26] observation of frustration among peer workers when progress toward abstinence was slow. Both studies also highlight a shift in understanding over time; Peer Navigators moved from viewing harm reduction as a temporary strategy to recognising it as a comprehensive approach that fosters autonomy and relationship-building, an evolution similarly documented in Tookey and colleagues’ findings. This paper extends this discussion by highlighting the personal transformation Peer Navigators undergo as they reconcile their abstinence-based recovery with the broader harm reduction approach.

The importance of training and orientation in preparing peer workers for their roles is also emphasised in other studies [8, 12, 20], though, as outlined previously, there was significant imprecision across the literature regarding the types, duration, and quality of the training available to peer workers. Thus, while reinforcing the necessity of training for peer workers stated above, this paper provides an in-depth exploration of the subjective experiences of peer workers during training phases and offers their insights into the perceived relevance and practical application of the training. This contribution fills a gap in the literature, providing a nuanced understanding of how training impacts peer workers’ roles and perceived effectiveness.

Additionally, the paper resonates with existing research on organisational culture and its role in the acceptance and professional development of peer workers [15]. For example, working across multiple statutory and third sector sites, the Peer Navigators experienced different organisational cultures and, as a consequence, faced varying levels of acceptance and resistance. However, while such resistance was noted in other scholarly works, such as, issues integrating into existing organisations, role constraints, and supervision by non-peer colleagues [11, 20], the SHARPS Peer Navigators described significant autonomy and flexibility in their roles. Resistance to the Peer Navigator intervention was therefore distinct and tended to concern organisational expectations and perceptions that Peer Navigator role boundaries encroached upon their roles. Moreover, this did not appear to be linked to professional versus lived experience knowledges [15, 29]. Rather, the novel intervention, with attendant local and study team support, represented a threat to existing practices and service structures, as evidenced through primary research data, both here and in other SHARPS publications [4]. This additional layer of complexity enriches the discourse around the tensions between experiential and professional knowledges, especially where elements of the former may engender emerging best practice, offering actionable insights for organisations seeking to improve the integration of peer workers into their workforces.

The literature also underscores the role of relationship building in the effectiveness of peer work [12, 29, 30], particularly as it relates to mentorship for generating positive client outcomes [8, 10]. In alignment with this, this paper highlights the capacity of peer workers to establish trusting relationships with their clients, sometimes in a single conversation. This finding reinforces arguments arising from previous evidence justifying peer workers as essential for supporting those ‘hardly reached’ by traditional approaches [31]. Work by Olding and colleagues [16] across four peer-staffed supervised drug consumption facilities in Vancouver, Canada, emphasised the value of support provided to peer workers by their (non-peer) colleagues. Consistent with this idea, the SHARPS study Peer Navigators described feeling ‘well looked after’ by the study team and most line managers across host services and intervention sites, analogous to the ‘committed family’ ethos described by Annand and colleagues’ [8] peer worker participants. Whilst the Peer Navigators faced similar challenges around peer worker acceptance from external organisations [11, 20] the design of the SHARPS intervention necessitated such Peer Navigator–inter-organisational interactions, rather than seeking to minimise or mitigate negative effects [20]. Thus, the experiences of the Peer Navigators add to the literature a more comprehensive understanding of the challenges peer workers face in garnering respect and demonstrating their value among colleagues across organisational boundaries. This offers a more comprehensive understanding of the relational dynamics that peer workers navigate in the workplace.

Co-workers (non-peer colleagues) have been recognised as an important resource for mutual support, given their understanding of the distinctive challenges faced in delivering services to people who are experiencing homelessness and/or use substances [16]. However, some peer workers expressed feeling undervalued and not taken seriously by their colleagues and other professionals they encountered in their work [11]. Highlighting this as a key challenge faced by peer workers in an overdose prevention setting, Mamdani et al. [20] recommended the addition of a designated staff member to assist peer workers in building relationships with external providers for facilitating client referrals. Similarly, peer workers in another study emphasised the value of internal organisational support networks in alleviating personal pressures and enabling further outcomes, such as paid employment [8]. Speaking also to organisational acceptance and support of peer work, Annand and colleagues’ [8] participants described their organisation as a committed and ethical ‘family’ dedicated to the mission of alleviating homelessness, a sentiment that promoted a unity of vision among the workforce. However, discussion of professional network development sufficient to support the spanning of organisational boundaries to facilitate effective peer work was limited across the reviewed literature, indicating a need for further exploration in this area.

Finally, while existing literature acknowledges the diverse training needs of peer workers, it often stops short of discussing career progression and advancement [16, 20]. This paper addresses this gap by exploring not just the current roles and training needs of peer workers, but also their career trajectories several years after the study ended. The paper provides new insight into the types of organisational support structures that facilitate these transitions, thereby filling a void in the current body of work. In summary, the present paper provides a more holistic view of the peer worker experience, enriching the literature from initial training to career progression. These insights have significant implications for refining support systems, enhancing service delivery, and fostering the professional growth of peer workers within organisations.

### Recommendations for services employing peer workers

Based on the comprehensive analysis of the findings and reflections from the peer workers during and after the SHARPS intervention, several recommendations emerge to enhance the effectiveness of peer workers and support their personal and professional growth. These recommendations integrate insights from all themes, making sense of the role, building capacity and adapting to organisational cultures, managing relationships and building professional networks, professional development opportunities, and from authors’ personal reflections upon their time as Peer Navigators and since the post came to an end.

### Provide comprehensive training and support for skill development

#### Recommendation

Invest in extensive training programmes that cover both interpersonal (‘soft’) skills and practical competencies, equipping peer workers with the tools necessary for their roles and future career advancement.

#### Rationale

Peer workers benefitted significantly from training that enhanced their communication, empathy, teamwork, and problem-solving abilities. Developing transferable skills was pivotal in shaping their career trajectories.

#### Implementation

- Design training curricula that include modules on active listening, empathy, conflict resolution, and teamwork.
- Offer practical skills training in areas like time management, case management, and harm reduction practices.
- Provide opportunities for peer workers to attend external courses, workshops, and pursue further education relevant to their roles.
- Encourage participation in activities that build confidence, such as public speaking engagements and facilitating meetings.

### Support personal growth and overcoming challenges

#### Recommendation

Recognise and address personal challenges such as social anxiety and imposter syndrome by providing mentorship and supportive environments, helping peer workers build resilience.

#### Rationale

Experiencing feelings of imposter syndrome was common among peer workers, but overcoming these challenges helped them adapt and manage similar feelings in subsequent roles. This resilience is crucial for career advancement.

#### Implementation

- Establish mentorship programmes pairing peer workers with experienced professionals (including longer-serving peer workers) who can provide guidance and encouragement.
- Create a supportive organisational culture that acknowledges and normalises feelings of self-doubt, offering resources to address them.
- Facilitate reflective practices and discussions that allow peer workers to share experiences and strategies for overcoming personal challenges.

### Provide challenging and diverse responsibilities to enhance confidence

#### Recommendation

Design peer roles that include varied and demanding tasks, supporting peer workers in stepping outside their comfort zones to foster professional growth and enhance confidence.

#### Rationale

Engaging in challenging aspects of the role—such as client advocacy, leading multidisciplinary team meetings, and speaking at conferences—increased peer workers’ confidence and prepared them for future professional positions.

#### Implementation

- Assign responsibilities that encourage peer workers to develop new skills and take on leadership roles.
- Support peer workers in participating in conferences, presentations, and collaborative projects.
- Provide opportunities for peer workers to lead initiatives or projects within the organisation.

### Encourage professional networking and collaboration

#### Recommendation

Facilitate networking opportunities for peer workers, recognising that building professional relationships may open doors for both individual advancement and organisational partnerships.

#### Rationale

Leveraging professional connections was instrumental in peer workers’ career development, leading to opportunities such as conference presentations, lectures, and collaborative research projects.

#### Implementation

- Introduce peer workers to key stakeholders and encourage attendance at networking events.
- Support peer workers in representing the organisation at external events and collaborations.
- Recognise and reward efforts to build professional networks that benefit both the individual and the organisation.

### Foster strong client relationships through empathy and compassion

#### Recommendation

Emphasise the importance of building meaningful relationships with clients, grounded in empathy, compassion, and respect.

#### Rationale

Peer workers found that strong relationships with clients were central to successful interventions, decreasing resistance and promoting positive change.

#### Implementation

- Provide training on empathetic communication and relationship-building techniques.
- Encourage peer workers to adopt asset-based approaches that focus on clients’ strengths and interests.
- Support peer workers in practicing active listening and ‘holding space’ for clients in emotionally charged situations.

### Encourage reflective practice and continuous learning

#### Recommendation

Support and facilitate reflective practice among peer workers to promote continuous learning and improvement.

#### Rationale

Engagement in reflective practice enhanced peer workers’ ability to deliver person-centred care and adapt to clients’ needs, particularly those continuing in specialist support roles.

#### Implementation

- Schedule regular supervision and reflective sessions where peer workers can discuss experiences and insights.
- Provide resources such as journals or guided reflection tools to facilitate personal growth.
- Encourage peer workers to set personal and professional development goals and support them in achieving these objectives.

### Clarify roles and responsibilities while promoting flexibility

#### Recommendation

Offer clear role definitions to help peer workers understand their responsibilities and boundaries while allowing flexibility to meet client needs.

#### Rationale

Peer workers initially struggled with role ambiguity, feeling like imposters and grappling with blurred boundaries. Clear definitions help alleviate confusion and build confidence.

#### Implementation

- Develop comprehensive job descriptions outlining specific duties, expectations, and limits of the peer worker role.
- Discuss roles during orientation and provide written guidelines on professional conduct and boundaries.
- Encourage flexibility in approach while maintaining adherence to organisational policies and ethical standards.

### Promote organisational acceptance and support for the peer worker role

#### Recommendation

Work towards securing buy-in from all levels of the organisation and partner agencies to minimise resistance and enhance collaboration.

#### Rationale

Peer workers faced resistance from some professionals who questioned the value of their role, impacting their effectiveness.

#### Implementation

- Educate all staff about the peer worker role, its benefits, and successes through presentations, workshops, and informational materials.
- Highlight positive outcomes and success stories associated with peer-led interventions.
- Encourage leadership to endorse and integrate the peer worker role within organisational structures and strategies.

### Embrace and embed harm reduction principles

#### Recommendation

Adopt harm reduction philosophies within the organisational framework and ensure alignment with peer workers’ approaches.

#### Rationale

Peer workers evolved to fully embrace harm reduction, recognising its value in supporting client autonomy and building relationships.

#### Implementation

- Provide comprehensive training on harm reduction principles to all staff.
- Incorporate harm reduction strategies into organisational policies, procedures, and service delivery models.
- Encourage open dialogue about harm reduction to address misconceptions and promote understanding.

### Facilitate career advancement opportunities

#### Recommendation

Create pathways for peer workers to progress in their careers, acknowledging their contributions and supporting their aspirations.

#### Rationale

Developing transferable skills and leveraging professional connections were pivotal in shaping peer workers’ career trajectories, enhancing job satisfaction and retention.

#### Implementation

- Offer professional development opportunities, such as advanced training or education programmes.
- Encourage peer workers to take on leadership roles or additional responsibilities that align with their career goals.
- Provide support in pursuing further education or qualifications relevant to their field.

## Conclusions

This study provides a nuanced exploration of peer workers’ experiences as they transitioned into and through their roles within the SHARPS intervention. Key themes identified were: the evolving understanding of their multifaceted roles; the importance of building capacity and adapting to organisational cultures; the critical role of managing relationships and professional networks; and the significance of professional development opportunities.

Peer workers navigated complex expectations and organisational dynamics, grappling with role definition and boundary setting while maintaining flexibility to meet clients’ diverse needs. Their journey highlighted the interplay between personal growth, skill development, and adaptability to varying organisational cultures. Adopting harm reduction principles emerged as transformative, enhancing their ability to support clients effectively.

The reflections of the Peer Navigators underscore the impact of comprehensive training, supportive environments, and opportunities for advancement. These insights informed recommendations aimed at enhancing the integration and effectiveness of peer workers within services. By addressing skill development, personal growth, role clarity, organisational support, and career progression, these recommendations provide a strategic framework for organisations seeking to maximise peer workers’ contributions.

Implementing these recommendations can lead to more effective peer-led interventions, improved client outcomes, and enriched professional experiences for peer workers. The findings emphasise the value of investing in peer workers as integral team members, capable of bridging service gaps and fostering positive change within communities.

This study contributes to the literature on peer work by illuminating the challenges and opportunities inherent in these roles. It highlights the necessity for organisational commitment to support peer workers through structured training, clear role definitions, and pathways for advancement. By embracing these strategies, organisations can harness peer workers’ unique strengths, ultimately enhancing service delivery and promoting better health and social outcomes for clients.

## Electronic supplementary material

Supplementary Material 1

Supplementary Material 2

## Data Availability

The datasets generated and/or analysed during the study are not publicly available. Individual privacy could be compromised if the dataset is shared due to the small sample involved.
