# Supplementary material for: Navigating transitions into, through, and beyond peer worker roles: insider insights from the Supporting Harm Reduction through Peer Support (SHARPS) study
Source: Harm Reduct J. 2024 Oct 28;21:191. doi: 10.1186/s12954-024-01109-4 (PMC11514757; doi:10.1186/s12954-024-01109-4)
Supplement: Supplementary file 1 — Supplementary Material 1 [file 12954_2024_1109_MOESM1_ESM.docx]

**Interview topic guides**

**Peer Navigators**

Interview 1 (pre-intervention)

- First week- how has it been? What's gone well/less well/ok? First impressions- the study, the team members that you've met, the Salvation Army, the stakeholder organisations you’ve met so far, the academic subject area (problem substance use and homelessness mainly), the university.
- Experiences of seminar and SDF event- atmosphere at these, interactions, topics covered- overall feeling and reflections, any surprises, any challenges, anything a bit tricky?
- Thoughts on the study overall- study background, approach, aims? Do you see any challenges or issues- if so, what are they?
- Intervention/manual development- how did you find the day overall? What are the key elements we need to include/think about in your view? Do we have any challenges associated with manualising? If 'yes', what are they? Anything else?
- Peer Navigator role in the study- what do you see your role as being? Thoughts on this? Aims and any concerns? Comprehensive induction and training planned, and it's early days- but anything that sticks out at this stage that’s missing or not enough that would support you in your role or help your learning/development? Anything that you want to know more about, or do that would help you with your work?
- Any questions or comments? Anything else?

Interview 2 (mid-intervention)

- Experiences in post so far
- Recruitment of participants – any challenges?
- Health checks and outcome measures – how are these going?
- Training, support and supervision – is there anything you need?
- Fidelity – are you doing what is expected as part of the intervention? Are you being asked to do things outwith your role?
- Acceptability - is the intervention/Peer Navigator acceptable to other staff/service users?
- Local context- accessibility of services, any barriers?
- Staff relationships and dynamic – any challenges?
- Suggestions for ways to improve intervention
- Any other challenges?

Additional Peer Navigator interview

- General discussion
- Experiences of retaining participants – working day to day with caseload
- Experiences of participant monies fund – usage, issues, access etc.
- Paired working – views on working with Peer Navigator in service, what would be ideal set up etc.
- Anything else wishing to raise, discuss, ask.

Final interview

- Reflection on intervention/post overall – things enjoyed, things that have been ok, challenges etc.
- Recruitment and retention of participants – experiences, challenges, suggestions for improvements
- Experience of conducting health checks and outcome measures, including change in planned approach. How long on average does it take to arrange the measures (taking account of texts, calls often repeated)?
- Training, support and supervision – anything you wanted to receive? Was support appropriate and sufficient? Were there things we should have told you before you started that we didn’t? Support: WhatsApp group, support from Jason, supervision from Adam, support from Service Managers and study team.
- How about preparing the settings? How was that? What could or should we have done to prepare them better, if anything?
- Fidelity – do you think the intervention/your role went as expected? Were you expected to do things beyond your role? Do you think you delivered the intervention the same as/similarly to the other three Peer Navigators? Why do you think this? Please expand. Is this an intervention that should be delivered exactly the same by each Peer Navigator? Risks of not? Benefits of not?
- Acceptability – is the intervention/Peer Navigator acceptable to other staff? How about to the service users?
- Support for participants – can you give some examples of what you did to support participants? To show breadth of what you do (and your hard work!).
- How well did the practical support monies work out? Any changes we should do here?
- Experience of being part of a research study – hard to disentangle from role of course, but the requirements of being connected to a study e.g. interviews, arranging and supporting the measures, filling in various logs
- If we were doing study again, would you make any changes? If so, what would they be?
- And relatedly, overall do you think the study is feasible to be rolled out further? To help answer, what worked really well? What worked less well? Operationally how could it have been improved upon?
- Thinking about a potential next stage study, do you see this as being able to be applied in other settings? Within the area and beyond e.g. social work, hospitals.
- Based on your experience and related to above, what would your thoughts/feelings be about randomising this intervention?
- Next steps – what are you doing next and why? Or what are you wanting to do next and why? Decision making underlying this e.g. wanted similar role, wanted different role
- Would you recommend the role to someone in your position (your position 18 months ago)?
- Anything else? Questions, comments, feedback
