# Supplementary material for: Navigating transitions into, through, and beyond peer worker roles: insider insights from the Supporting Harm Reduction through Peer Support (SHARPS) study
Source: Harm Reduct J. 2024 Oct 28;21:191. doi: 10.1186/s12954-024-01109-4 (PMC11514757; doi:10.1186/s12954-024-01109-4)

**
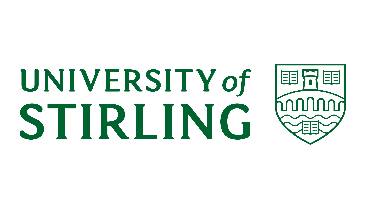
**

**Supporting Harm Reduction through Peer Support (SHARPS):** **Testing the feasibility and acceptability of a peer-delivered, relational intervention for people with problem substance use who are homeless, to improve health outcomes, quality of life and social functioning, and reduce harms**

**Peer Navigators - Reflective Diaries Guide**

We have invited you to keep a reflective diary in the run-up to the intervention and during it. The participant information sheet we have given you provides more information on this.

The main purpose of these diaries is to capture your reflections on the intervention and your role in it. Please remember not to reveal any personal/identifiable information about others. Please also remember that you are free to share as much or as little as you like from your diaries with the study team - what you share is your choice. Please don’t hesitate to speak to Rebecca, Tessa or Hannah if you have any questions about this, or if you change your mind about keeping a diary.

To help guide you in these reflections, we have created a guide with some prompts. Please do not feel limited to these! Some will be more relevant at different stages of the role.

- How you are findings your job and your role overall
- How you are finding the Salvation Army induction, background reading for the study etc
- How you found the early meetings with the study team and those on the intervention development day
- Were any support needs not met in the early days and weeks?
- How you are finding the training you’ve been going on
- How you are finding being in your local services and your relationship with your Service Manager
- What your impressions are of the services you’re working in
- How are people responding to you in the host services you are in and external to services
- How are people responding to the idea of the Peer Navigators or the SHARPS project?
- How have you experienced any visits you’ve done locally
- How you are feeling about starting the intervention in October
- How your relationships are developing with other staff members in your host service and other services
- How it’s going with service users/clients in your local services and connections you are making
- How you are getting on with the other Peer Navigators
- How useful the Whats App group is for peer support
- Anything that you’re finding particularly good or particularly tricky, anything that is surprising you - things of note
- How the recruitment of participants is going
- How the intervention is going overall, and how it’s going with each participant
- Your thoughts and feelings about coming to the end of the intervention and looking ahead.


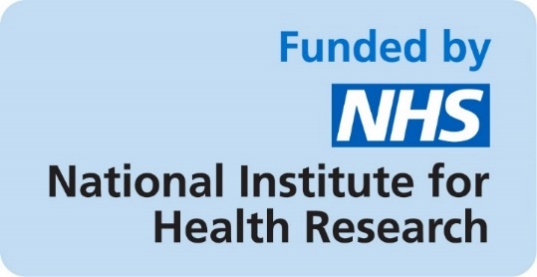

Supplement: Supplementary file 2 — Supplementary Material 2 [file 12954_2024_1109_MOESM2_ESM.docx]
